# A high calcium diet containing nonfat dry milk reduces weight gain and associated adipose tissue inflammation in diet-induced obese mice when compared to high calcium alone

**DOI:** 10.1186/1743-7075-9-3

**Published:** 2012-01-23

**Authors:** Anthony P Thomas, Tamara N Dunn, Josephine B Drayton, Pieter J Oort, Sean H Adams

**Affiliations:** 1 Department of Nutrition, University of California, One Shields Avenue, Davis, CA, USA; 2 Department of Animal Science, University of California, One Shields Ave., Davis, CA 95616, USA; 3 Obesity & Metabolism Research Unit, United States Department of Agriculture-Agricultural Research Service Western Human Nutrition Research Center, 430 W. Health Sciences Dr., Davis, CA 95616, USA

**Keywords:** Calcitriol, calcium, dairy, obesity, inflammation

## Abstract

**Background:**

High dietary calcium (Ca) is reported to have anti-obesity and anti-inflammatory properties. Evidence for these properties of dietary Ca in animal models of polygenic obesity have been confounded by the inclusion of dairy food components in experimental diets; thus, effect of Ca *per se *could not be deciphered. Furthermore, potential anti-inflammatory actions of Ca *in vivo *could not be dissociated from reduced adiposity.

**Methods:**

We characterized adiposity along with metabolic and inflammatory phenotypes in diet-induced obese (DIO) mice fed 1 of 3 high fat diets (45% energy) for 12 wk: control (*n *= 29), high-Ca (*n *= 30), or high-Ca + nonfat dry milk (NFDM) (*n *= 30).

**Results:**

Mice fed high-Ca + NFDM had reduced body weight and adiposity compared to high-Ca mice (*P *< 0.001). Surprisingly, the high-Ca mice had increased adiposity compared to lower-Ca controls (*P *< 0.001). Hyperphagia and increased feed efficiency contributed to obesity development in high-Ca mice, in contrast to NFDM mice that displayed significantly reduced weight gain despite higher energy intake compared to controls (P < 0.001). mRNA markers of macrophages (e.g., CD68, CD11d) strongly correlated with body weight in all diet treatment groups, and most treatment differences in WAT inflammatory factor mRNA abundances were lost when controlling for body weight gain as a covariate.

**Conclusions:**

The results indicate that high dietary Ca is not sufficient to dampen obesity-related phenotypes in DIO mice, and in fact exacerbates weight gain and hyperphagia. The data further suggest that putative anti-obesity properties of dairy emanate from food components beyond Ca.

## Background

Epidemiological or cross-sectional studies in human populations support an inverse relationship between dietary calcium (Ca) and dairy food consumption with obesity. Several clinical weight loss intervention studies that included high-Ca or dairy foods indicated a positive effect on body fat loss [1,2], but this has not been universally observed [3], suggesting that these effects are context-specific (e.g., dependent upon the specific population, dietary Ca status, type of dairy food, or provision of elemental Ca vs. Ca in a dairy matrix). In contrast, fat loss or reduced weight gain in response to high-Ca plus dairy protein in rodent obesity models has been consistently reported [4-7]. The mechanisms underlying the metabolic effects of Ca and dairy foods to reduce adiposity remain to be elucidated. Increased fecal fat loss due to the formation of indigestible Ca soaps in the gastrointestinal tract has been proposed as a possible mechanism by which high dietary Ca reduces adiposity [8]. Research from one group has provided evidence *in vitro *and in the aP2-agouti transgenic mouse model of diet-induced obesity for a potential role of the calcitrophic hormone 1,25-dihydroxyvitamin D (calcitriol) in promoting adipocyte lipogenesis and inhibiting lipolysis, thus encouraging adipocyte lipid accumulation [9-11]. According to this model, increasing dietary Ca would suppress circulating calcitriol, discouraging energy storage and white adipose tissue (WAT) expansion.

Dietary Ca may also impact inflammatory phenotypes associated with obesity. Chronic inflammation of WAT, especially in visceral depots, contributes to the pathogenesis of obesity-related insulin resistance [12-14], and WAT macrophages are a prominent source of pro-inflammatory cytokine production [15-18]. Calcitriol increased pro-inflammatory cytokine gene expression and secretion from cultured adipocytes and macrophages, and this response was dependent on intracellular Ca-provoked reactive oxygen species (ROS) generation; co-culture of both cell types increased gene expression and protein secretion of pro-inflammatory cytokines, which was further enhanced by calcitriol [19]. *In vivo *evidence for anti-inflammatory properties of high dietary Ca was demonstrated in the aP2-agouti transgenic mouse obesity model in which increased dietary Ca was associated with reduced plasma calcitriol [20,21]. Despite a potential mechanism for direct anti-inflammatory properties of dietary Ca through suppression of circulating calcitriol, reduced inflammation could not be dissociated from lower body weight and adiposity in studies of the aP2-agouti transgenic mice. Furthermore, one cannot exclude a confounding effect of adipose agouti protein overexpression on these phenotypes, or possible effects of agouti expression in macrophages since the aP2 promoter can be activated in both macrophages and fat cells. Thus, further research is necessary to confirm potential anti-inflammatory properties of high dietary Ca and/or dairy in polygenic obesity models while controlling for body weight and adiposity changes typically observed in animals fed these diets.

Additional bioactive components of dairy foods (e.g. branched chain amino acids, peptide fragments with angiotensin converting enzyme (ACE)-inhibitory properties) might contribute to anti-obesity/anti-inflammatory outcomes, since high Ca in a dairy matrix yielded greater effects vs. elemental Ca alone in aP2-agouti mice [22]. Studies demonstrating an anti-obesity effect of increased dietary Ca in polygenic obesity models have been only in the context of nonfat dry milk (NFDM) or whey providing all dietary protein [4,6,7]. Thus, comparing high Ca diet in matrices containing differing protein and carbohydrate sources (dairy and non-dairy) is of interest to understand if non-Ca factors in dairy foods could play a role in regulation of metabolism and obesity-associated inflammation.

To address whether dietary Ca can impact obesity phenotypes and to understand if putative anti-inflammatory effects of high-Ca or dairy-based diets are yoked to body weight, we investigated the potential anti-obesity and anti-inflammatory effects of high dietary Ca in a well-characterized polygenic mouse model of diet-induced obesity (DIO): male C57BL/6J mice display susceptibility to diet-induced obesity and glucose intolerance when fed a high fat diet [23] and we have previously described obesity with associated inflammation and impaired glucose homeostasis when feeding these mice a diet providing 45% energy as fat (with soy- and sucrose/cornstarch-based protein and carbohydrate sources, respectively) for up to 12 wk [24]. We hypothesized that independent of differences in body weight gain, DIO mice fed high-Ca would have reduced body weight, decreased adiposity, and improved metabolic and inflammatory phenotypes compared to control mice fed a lower Ca diet. Our study design also enabled a comparison of metabolic and inflammatory outcomes in high-Ca DIO mice relative to animals fed high-Ca in a dairy matrix (NFDM as the sole protein source and containing primarily dairy-based carbohydrates).

## Materials and methods

### DIO Mice

All mouse protocols were approved by the University of California at Davis Institutional Animal Care and Use Committee according to Animal Welfare Act guidelines. Four-wk-old male C57BL/6J mice were purchased from the Jackson Laboratory and individually housed under standard temperature (20-22°C) and light/dark cycle (12 h:12 h) conditions in a pathogen-free facility. Individually-housed mice were fed Picolab^® ^Mouse Diet 20 (Purina LabDiet^®^) providing ~22, ~22, and ~56% energy as protein, fat, and carbohydrate, respectively, for a 1 wk acclimation period. Weight matched mice were randomly assigned to purified experimental diets containing 45% energy as fat (Table 1) for 12 wk. Treatment groups were: soy protein-based control (0.5% Ca, *n *= 29; Control), soy protein-based high-Ca (1.5% Ca, *n *= 30; high-Ca), or high-Ca in the context of nonfat dry milk protein and carbohydrates (1.5% Ca, *n *= 30; high-Ca + NFDM). In the case of the NFDM diet, to ensure equal macronutrient energy contribution compared to the other diets while maintaining a dairy nutrient matrix, protein (casein and whey-derived lactalbumin) and carbohydrate (galactose + glucose, the components of lactose) were added. Ca phosphate (CaPO_4_) was added to the soy-protein based diets to match that naturally-derived from NFDM (to control for type of dietary Ca), with Ca carbonate providing all additional Ca to the experimental diets as typically used in rodent studies comparing different dietary Ca. Cellulose content was adjusted accordingly to account for added Ca in the high-Ca diets to ensure matched macro and micronutrient content of diets, and amounts of fiber are all within the normal range used in purified mouse diets. The control diet and 12 wk timeframe were previously shown to elicit obesity, increased WAT inflammation, and insulin resistance in this model [24]. Mice were given free access to food and water with body weight and food intake (plus spillage) measurements made every 2-3 d. Fecal collections over 48 h were made on a subset of randomly chosen mice (*n *= 10/group) at wk 10. Feces were weighed and stored at -80°C until bomb calorimetry was performed by Covance Laboratories. Percent fecal energy loss was calculated from each animal's 48 h fecal energy loss and energy consumption. Glucose tolerance tests were administered on a subset of randomly chosen mice (*n *= 15-16/group) as described elsewhere [24]. At wk 12, mice were briefly food-deprived prior to tissue and blood collection as described in detail [24].

**Table 1:** Mouse Diet Composition*

	Diet [product code]		
	**Control (0.5% Ca) [TD.08511]**	**High-Ca (1.5% Ca) [TD.09068]**	**High-Ca + NFDM [TD.09069]**

**Ingredients (g/kg)**			
Isolated Soy Protein	195	195	0
Milk Powder, skim	0	0	400
Casein	0	0	29.5
Lactalbumin	0	0	7.5
DL-Methionine	2.34	2.34	1.24
L-Cystine	1	1	0.55
Sucrose	190	190	0
Galactose	0	0	18.5
Dextrose, monohydrate	0	0	20.29
Corn Starch	87.99	87.99	0
Maltodextrin	200	200	200
Cellulose	50	25.25	40
Lard	205	205	205
Soybean Oil	20	20	20
Mineral Mix w/o Ca & P (98057)	17	17	17
Calcium Phosphate, dibasic	14.5	14.5	0
Calcium Carbonate	1.1	26	24.5
Vitamin Mix, AIN-93-VX (94047)	12.7	12.7	12.7
Choline Bitartrate	3.2	3.2	3.2
TBHQ, antioxidant	0.02	0.02	0.02
**Macronutrients (% by weight)**			
Protein	17.3	17.3	17.2
Carbohydrate	47.4	47.4	44.4
Fat	23.4	23.4	22.8
**Macronutrients (% kcal)**			
Protein	14.7	14.7	15.2
Carbohydrate	40.4	40.4	39.3
Fat	44.9	44.9	45.4
**KJ/g**	19.7	19.7	18.8

*Diets formulated and produced by Harlan Teklad. NFDM, nonfat dry milk.

### Total RNA Isolation and Gene Expression Analyses

Total RNA was isolated from whole retroperitoneal (RP) fat pads, cDNA was prepared, and 384-well quantitative PCR utilizing gene-specific Taqman^® ^primers and FAM-MGB labeled probes (Additional file 1; Assays-on-Demand^®^, Applied Biosystems) was conducted as previously described in detail [24].

### Plasma Cytokines/Chemokines, Glucose, Insulin, and Calcitriol

Plasma cytokines/chemokines were measured according to manufacturer's instructions in duplicate for plasma samples (20 μL/reaction; *n *= 15/group) using a Milliplex^® ^MAP Mouse Cytokine/Chemokine Kit (Millipore) on a Bio-Plex system with xMAP^® ^Luminex^® ^technology utilizing Bio-Plex Manager version 5.0 software (Bio-Rad). Plasma glucose was measured according to manufacturer's instructions in duplicate (5 μL/reaction) with the Glucose (HK) Assay Kit (Sigma). Plasma insulin was measured according to manufacturer's instructions in duplicate (5 μL/reaction) with an Ultra Sensitive Mouse Insulin ELISA Kit (Crystal Chem). Plasma (100 μL pooled plasma from 3 mice/reaction) calcitriol levels (*n *= 8-9/group) were measured by 1,25-dihydroxyvitamin D radioimmunoassay (Immunodiagnostic Systems Inc.) according to manufacturer's instructions.

### Western Blot Analysis of Brown Adipose Tissue Uncoupling Protein 1 (UCP1)

Whole intrascapular brown adipose tissue (BAT) pads (*n *= 8/group) were homogenized in M-PER^® ^Reagent (200 μL/100 g tissue) in the presence of HALT Protease and Phosphatase Inhibitor Cocktail + 0.5 M EDTA (Pierce). Proteins (25 μg) were separated using a 12% Bis-Tris Criterion™ XT Precast Gel (Bio-Rad) and NuPAGE^® ^MOPS SDS Running Buffer (Invitrogen) at 200 V for ~ 1 h. Proteins were transferred at 30 V for 1 h to an Immobilon™ polyvinylidene fluoride membrane (Millipore), blocked overnight at 4°C with 5% nonfat dry milk in PBS with 0.1% Tween 20 (PBS-T) and incubated with rabbit polyclonal anti-UCP1 primary antibody (1:5000 dilution; U6382, Sigma) at room temperature for 1 h followed by 3 × 15 min washes in PBS-T. The membrane was incubated with HRP-conjugated goat anti-mouse IgG secondary antibody (1:10000 dilution; 1031-05, Southern Biotech) at room temperature for 1 h followed by 3 × 5-15 min washes in PBS-T. Antibody binding was visualized and relative protein levels were quantified using chemiluminescence (Immun-Star™ WesternC™ Chemiluminescent Kit, Bio-Rad) and ChemiDoc™ XRS+ Imaging System (Bio-Rad). Membrane was stripped using Restore™ PLUS Western Blot Stripping Buffer (Pierce) and reprobed using a pig monoclonal anti-glyceraldehyde-3-phosphate dehydrogenase (GAPDH) antibody (1:1000 dilution; NB300-221, Novus Biologicals) to control for protein loading.

### Liver Triglycerides

Liver lipid extraction (*n *= 10/group) was performed utilizing a modified Folch method [25]. Briefly, approximately 100 mg of liver was homogenized in a 2:1 (v:v) chloroform:methanol solution (20 μL/mg tissue) followed by separation of the organic phase and dehydration by speed-vac prior to being reconstituted in 1 mL isopropanol. Triglyceride content of lipid extracts was measured using an enzymatic assay kit (TR0100, Sigma) according to manufacture's instructions.

### Statistical Analysis

A one-way ANOVA with Newman-Keuls Multiple Comparison *post hoc *test was used for three-group comparisons (Prism v. 4.0, Graphpad). Two-way repeated measures ANOVA was used to assess effects of diet, time, and diet × time interactions on body weight gain and cumulative energy intake. Pearson's correlation coefficient was determined to assess bivariate relationships between body weight and CD68/CD11d mRNA abundance. In order to assess the contribution of differences in weight gain on markers of RP-WAT macrophage infiltration and inflammation, and to assess the impact of food intake on weight gain, ANCOVA was used to compare the group means of the log transformed outcome variables controlling for the indicated covariate (SAS for Windows Release 9.2). Data are expressed as mean ± SEM and differences considered to be statistically significant at *P *≤ 0.05.

## Results

### Body Weight Gain, Cumulative Energy Intake, and Feed Efficiency

**Figure 1:**
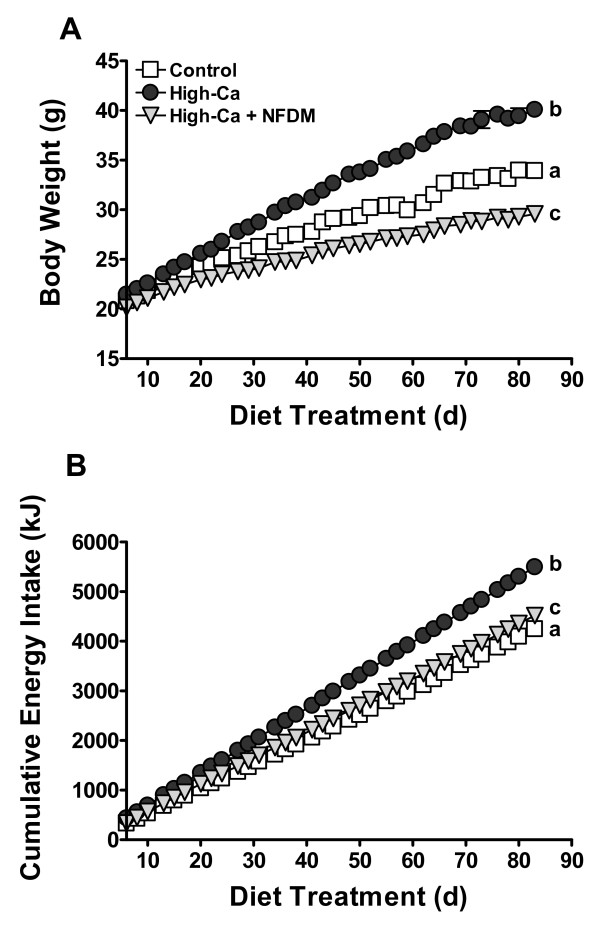
**Body weights (A) and cumulative energy intake (B) in DIO mice fed 0.5% Ca (Control), 1.5% Ca (High-Ca), or 1.5% Ca + nonfat dry milk (High-Ca + NFDM)**. Values are means ± SE, *n *= 29 (Control) or 30/treatment. Some error bars are within symbols. Treatments with different letters are significantly different (*P *< 0.05).

Body weight gain and terminal body weights were significantly reduced in mice fed high-Ca + NFDM relative to controls (Figure 1A). Contrary to our expectations, mice fed high-Ca were significantly heavier than controls (Figure 1A). There was a significant diet × time interaction on body weight gain (*P *< 0.0001). Compared to controls, mean body weight was heavier starting at d 27 (*P *< 0.05) and lower starting at d 36 (*P *< 0.01) of diet onward for mice fed high-Ca or high-Ca + NFDM, respectively. Mice fed high-Ca + NFDM weighed significantly less than mice fed high-Ca starting at d 17 (*P *< 0.05) of diet onward.

Increased cumulative energy intake in mice fed high-Ca compared to both controls and high-Ca + NFDM fed mice (Figure 1B) may account for increased body weight gain in this group, in contrast to high-Ca + NFDM mice that displayed greater cumulative energy intake compared to controls (Figure 1B) despite significantly lower body weight gain. Terminal body weights were not significantly different between mice fed high-Ca compared to controls when controlling for food intake (ANCOVA), indicating that intake explained much of the obesity in the high-Ca group; in contrast, when controlling for food intake, weight remained significantly reduced in mice fed high-Ca + NFDM compared to controls or mice fed high-Ca (*P *< 0.0001). There was a significant diet × time interaction on cumulative energy intake (*P *< 0.0001), with mean cumulative energy intake higher starting at d 13 (*P *< 0.05) and at d 57 (*P *< 0.05) of diet onward for mice fed high-Ca or high-Ca + NFDM, respectively, compared to controls. Feed efficiency (mg weight gain/kJ consumed) in mice fed high-Ca + NFDM was reduced (2.3 *+ *0.1) compared to controls (3.5 ± 0.1; *P *< 0.001) and high-Ca mice (3.8 ± 0.1; *P *< 0.001); the latter group had increased feed efficiency compared to controls (*P *< 0.01).

To determine the potential mechanisms underlying the marked differences in feed efficiencies, we examined markers of BAT activation considering the importance of this tissue to thermogenesis in mice. BAT weight was significantly increased in mice fed high-Ca compared to both controls and high-Ca + NFDM fed mice (Table 2). UCP1 protein content of BAT was increased to 1.7 fold of controls in mice fed high-Ca vs. the other groups (Additional file 2). Another factor potentially contributing to differences in feed efficiency is net energy absorption in the gut. There were significant diet-related differences in fecal output and fecal energy density (Additional file 3), and energy loss in the feces (as a % of energy intake) was significantly decreased and increased in mice fed high-Ca and high-Ca + NFDM, respectively (Additional file 3). Increased energy consumption coupled to reduced fecal energy loss resulted in significantly increased net metabolizable energy (in kJ/d) in mice fed high-Ca (66.2 ± 1.8; *P *< 0.001) compared to controls (48.1 ± 1.3) and high-Ca + NFDM mice (47.0 ± 1.4).

**Table 2:** Tissue weights in diet induced obese mice fed 0.5% Ca (Control), 1.5% Ca (High-Ca), or 1.5% Ca + nonfat dry milk (High-Ca + NFDM)^1^

	Diet		
**Tissue**	**Control**	**High-Ca**	**High-Ca + NFDM**

Liver (g)	1.2 ± 0.03^a^	1.7 ± 0.07^b^	1.1 ± 0.02^a, c^
Liver (% body weight)	3.5 ± 0.05^a^	4.1 ± 0.12^b^	3.7 ± 0.06^a, c^
Gastrocnemius muscles (mg)	301 ± 3.6^a^	311 ± 4.2^b^	288 ± 2.9^c^
Gastrocnemius muscles (% body weight)	0.9 + 0.01^a^	0.8 ± 0.02^b^	1.0 ± 0.01^c^
Brown adipose tissue (mg)	176 ± 8.3^a^	299 ± 12.1^b^	98 ± 4.1^c^
Brown adipose tissue (% body weight)	0.5 ± 0.02^a^	0.7 ± 0.02^b^	0.3 ± 0.01^c^
Spleen (mg)	73 ± 1.6^a^	86 ± 2.5^b^	66 ± 1.8^c^
Spleen (% body weight)	0.2 ± 0.00	0.2 ± 0.01	0.2 ± 0.01

^1^Values are means + SE, *n *= 29 or 30. Values with different superscript letter are significantly different (*P *< 0.05).

### Body Composition and Metabolic Phenotypes

**Figure 2:**
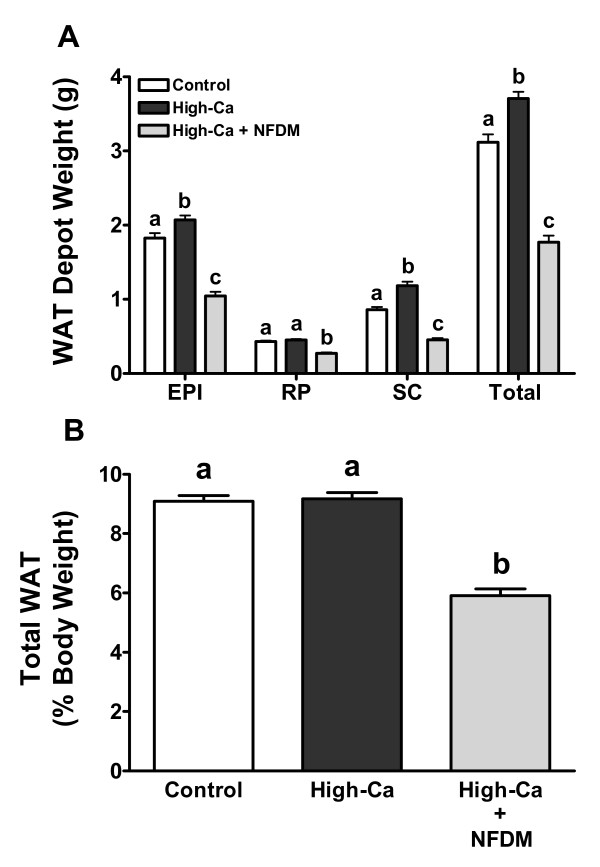
**Absolute (A) and relative (B) WAT depot weights in DIO mice fed 0.5% Ca (Control), 1.5% Ca (High-Ca), or 1.5% Ca + nonfat dry milk (High-Ca + NFDM)**. Values are means ± SE, *n *= 29 (Control) or 30/treatment. Treatments with different letters are significantly different (*P *< 0.05).

Absolute weights of retroperitoneal (RP), epididymal (EPI), and subcutaneous (SC) fat pads were all significantly lower in mice fed high-Ca + NFDM compared to controls and mice fed high-Ca (Figure 2A), which was reflected in reduced total % body fat (Figure 2B). Weights of EPI and SC fat pads were significantly heavier in high-Ca mice compared to controls (Figure 2A), but total % body fat was not different between these groups (Figure 2B). Liver weights were significantly heavier in high-Ca mice compared to both controls and mice fed high-Ca + NFDM (Table 2); the latter two groups did not differ significantly. This corresponded to increased liver triglycerides (mg/g) in mice fed high-Ca (98.4 ± 16.5) compared to controls (59.1 ± 9.9; *P *< 0.05) and high-Ca + NFDM mice (28.1 ± 10.1; *P *< 0.01).

**Figure 3:**
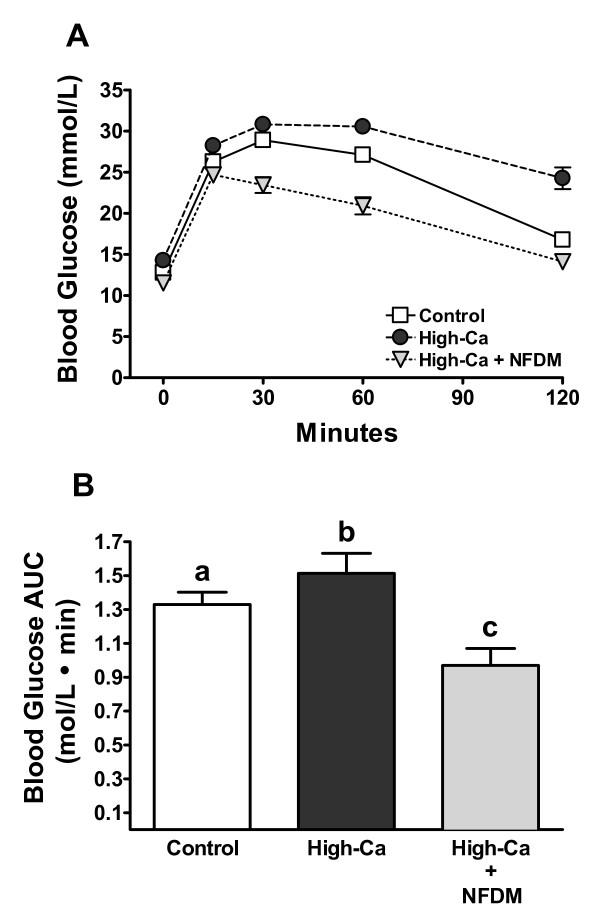
**Blood glucose concentrations (A) and AUC (B) in DIO mice fed 0.5% Ca (Control), 1.5% Ca (High-Ca), or 1.5% Ca + nonfat dry milk (High-Ca + NFDM) following an i.p. glucose tolerance test**. Values are means ± SE, *n *= 15 or 16/treatment. Some error bars are within symbols. Treatments with different letters are significantly different (*P *< 0.05). Fasting blood glucose was reduced in mice fed high-Ca + NFDM (11.5 ± 0.3 mmol/L) compared to DIO controls (12.8 ± 0.4 mmol/L; not significant) and mice fed high-Ca (14.3 ± 0.8 mmol/L; *P *< 0.01).

Mice fed high-Ca + NFDM had improved glucose tolerance (Figure 3A) with significantly reduced glucose area under the curve compared to controls and mice fed high-Ca (Figure 3B). Postabsorptive plasma glucose concentrations (mmol/L) measured at the time of tissue collection was significantly higher in mice fed high-Ca (20.6 ± 0.6; *P *< 0.001) and lower in high-Ca + NFDM fed mice (16.2 ± 0.4; *P *< 0.01), compared to controls (18.2 ± 0.4). Postabsorptive plasma insulin levels (pmol/L) followed the same trend: significantly higher in mice fed high-Ca (225.8 ± 7.7; *P *< 0.001) and significantly lower in mice fed high-Ca + NFDM (35.9 ± 3.5; *P *< 0.01) compared to controls (100.3 ± 7.75).

Dietary Ca-associated changes in expression of WAT fatty acid synthase (FAS) and interleukin 15 (IL-15) may regulate lipid accumulation, and shifts in uncoupling protein 2 (UCP2) could play a role in mitochondrial function and reactive oxygen species (ROS) generation [26]. FAS mRNA abundance in RP-WAT was not significantly different among diet treatment groups (Table 3); RP-WAT mRNA abundance of UCP2 was significantly decreased in mice fed high-Ca and high-Ca + NFDM fed mice compared to controls (Table 3), and this reduction was greater in mice fed high-Ca. mRNA abundance of IL-15 in RP-WAT was significantly decreased in mice fed high-Ca compared to controls (Table 3). IL-15 mRNA expression does not necessarily coincide with protein secretion [27] and skeletal muscle is the major site of IL-15 mRNA transcription and probably secretion [28,29]. We noted that plasma IL-15 levels were increased, although highly variable and not statistically significant, in mice fed high-Ca + NFDM compared to both controls and mice fed high-Ca (Table 4).

**Table 3:** RP-WAT mRNA levels in diet induced obese mice fed 0.5% Ca (Control), 1.5% Ca (High-Ca), or 1.5% Ca + nonfat dry milk (High-Ca + NFDM)^1^

	Diet		
**Gene**	**Control**	**High-Ca**	**High-Ca + NFDM**
	**% of Control**		

*Cd68*	100.0 ± 8.3^a^	213.1 ± 19.5^b^	70.9 ± 15.5^a^
*Itgad (CD11d)*	100.0 ± 13.1^a^	234.7 ± 28.5^b^	8.7 ± 2.5^c^
*Ccl2 (MCP-1)*	100.0 ± 9.2^a^	156.2 ± 9.0^b^	43.6 ± 4.7^c^
*Tnf (TNF*α)	100.0 ± 4.3^a^	154.3 ± 10.5^b^	65.4 ± 8.2^c^
*Il6*	100.0 ± 5.2^a^	127.1 ± 10.2^b^	75.7 ± 4.7^c^
*Hif1a (HIF-1α)*	100.0 ± 8.0^a^	82.3 ± 4.1^b^	51.2 ± 3.0^c^
*Il10*	100.0 ± 9.0^a^	263.6 ± 29.9^b^	97.0 ± 16.2^a^
*Retnla (FIZZ-1)*	100.0 ± 5.2^a^	174.9 ± 16.3^b^	78.9 ± 7.9^a^
*Calca (CGRPα)*	100.0 ± 15.3	75.5 ± 6.7	102.5 ± 13.8
*Il15*	100.0 ± 3.4^a^	68.7 ± 8.4^b^	94.6 ± 7.2^a^
*Fas*	100.0 ± 2.8	112.1 ± 4.7	105.1 ± 4.1
*Ucp2*	100.0 ± 6.0^a^	55.5 ± 4.4^b^	81.9 ± 4.6^c^

^1^Values are means + SE, *n *= 29 or 30. Values with different superscript letter are significantly different (*P *< 0.05).

**Table 4:** Plasma cytokines in diet induced obese mice fed 0.5% Ca (Control), 1.5% Ca (High-Ca), or 1.5% Ca + nonfat dry milk (High-Ca + NFDM)^1^

	Diet		
**Cytokine**	**Control**	**High-Ca**	**High-Ca + NFDM**
	**ng/L**		

IL-6	2.7 ± 0.5	4.1 ± 1.1	1.6 ± 0.3*
IL-10	3.7 ± 3.3	6.4 ± 4.4	3.9 ± 2.5
IL-12(p70)	4.3 ± 1.7	12.9 ± 4.2	7.9 ± 4.5
MCP-1	16.1 ± 2.5	16.1 ± 3.7	9.6 ± 3.9
TNFα	3.2 ± 0.2	3.0 ± 0.2	3.8 ± 0.9
IL-15	38.6 ± 6.2	48.2 ± 11.4	70.8 ± 22.7

^1^Values are means + SE, *n *= 15. *P < 0.05 compared to mice fed high-Ca.

### Inflammatory Phenotypes and Plasma Calcitriol

A suite of genes typically associated with obesity-induced WAT macrophage infiltration, inflammation, and hypoxic stress were surveyed to determine if dietary Ca or dairy reduce these parameters in DIO mice. We have previously measured this panel of markers and found them to be sensitive to DIO in this model [24]. RP-WAT mRNA abundance of the macrophage specific surface antigen CD68 was increased to ~200% and decreased to ~71% of control values in mice fed high-Ca and high-Ca + NFDM fed mice, respectively (Table 3). We have previously shown CD11d (leukocyte exclusive integrin α_D_β_2_) RP-WAT mRNA abundance was markedly increased obese mice relative to non-obese controls [24], and this parameter was increased to ~235% and decreased to ~9% of control values in high-Ca and high-Ca + NFDM fed mice, respectively (Table 2). Inflammatory markers associated with obese WAT (monocyte chemoattractant protein 1, MCP-1; tumor necrosis factor α, TNFα; IL-6) displayed significantly increased and decreased mRNA abundances in RP-WAT of mice fed high-Ca and high-Ca + NFDM, respectively, compared to controls (Table 3). However, hypoxia inducible factor 1α (HIF-1α) mRNA abundance in RP-WAT was significantly decreased in mice fed high-Ca or high-Ca + NFDM compared to controls (Table 3). RP-WAT mRNA abundance of IL-10, an anti-inflammatory cytokine, and found in inflammatory zone 1 (FIZZ-1), a marker of alternatively activated macrophages, were significantly increased in mice fed high-Ca compared to both controls and high-Ca + NFDM fed mice (Table 3). Macrophage-derived inflammatory cytokines have previously been shown to increase calcitonin gene related peptide α (CGRPα) expression in adipocytes and CGRPα may have anti-inflammatory effects on macrophages [30,31]. This target is also regulated in neurons and thyroid cells by Ca status and calcitriol [32,33]. However, RP-WAT mRNA abundance of CGRPα was not different between diet treatment groups (Table 3). Despite differences in visceral WAT inflammatory gene expression, differences in plasma markers associated with inflammation were less apparent and highly variable (Table 4).

**Figure 4:**
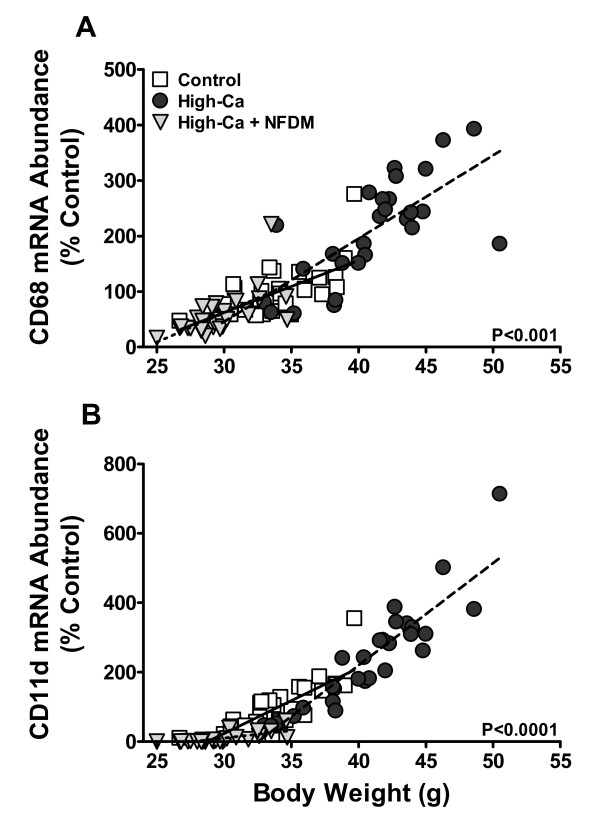
**Relative mRNA levels of CD68 (A) and CD11d (B) in RP-WAT are significantly correlated to body weight in DIO mice fed 0.5% Ca (Controls, *r *= 0.62), 1.5% Ca (High-Ca; *r *= 0.76), or 1.5% Ca + nonfat dry milk (High-Ca + NFDM; *r *= 0.63), *n *= 29 (Control) or 30/treatment**. P-values apply to all correlations within each panel.

Previous observations that high dietary Ca both with and without dairy reduce WAT inflammatory gene expression were confounded by Ca/dairy-associated reductions in body weight and adiposity [20,21]. To illustrate the relative influence of body weight on WAT macrophage accumulation, correlations between body weight and macrophage marker mRNA levels were examined (Figure 4). There were significant positive correlations between body weight and the mRNA expression of CD68 and CD11d in RP-WAT of DIO mice, regardless of diet treatment, and across a broad range of body weights (Figure 4). Controlling for body weight as a covariate, treatment differences in WAT CD68 mRNA abundances were not significantly different indicating that CD68-positive macrophage numbers in WAT are closely yoked to body weight. Controlling for weight and food intake, differences in CD11d mRNA abundance comparing controls and high-Ca mice were not statistically significant, but the reduction in CD11d expression in mice fed high-Ca + NFDM remained highly significant (*P *< 0.0001). RP-WAT mRNA abundances of other inflammatory markers (see Table 3) were not significantly different among diet treatment groups when controlling for body weight, with the exceptions of MCP-1 and HIF-1α: MCP-1 remained significantly decreased in high-Ca + NFDM mice compared to controls (*P *< 0.001) or high-Ca mice (*P *< 0.01), and HIF-1α remained significantly decreased in mice fed both of the high-Ca diets compared to controls (*P *< 0.01).

Calcitriol stimulates inflammatory responses in cultured adipocytes and macrophages [19], and dietary Ca suppression of circulating calcitriol was observed concurrent with reduced WAT inflammation associated with obesity development [20,21]. Plasma calcitriol (pmol/L) was significantly reduced in mice fed high-Ca (123.9 ± 13.0) compared to controls (187.1 ± 8.6; *P *< 0.01) and high-Ca + NFDM fed mice (168.9 ± 16.6; *P *< 0.05), but this was not associated with reductions in adiposity and inflammation. The more modest reduction in plasma calcitriol in mice fed high-Ca + NFDM was not significantly different compared to controls.

## Discussion

Consistent with previous reports [4,6,7,9], we showed reduced body weight and adiposity upon feeding a NFDM diet containing high Ca to high fat fed polygenic DIO rodents. Reductions in body weight and adiposity were accompanied by lower WAT inflammation and liver triglycerides as well as improved glucose tolerance. However, the data do not support the idea that high dietary Ca in isolation provides protection against obesity and associated inflammation since DIO mice fed a soy protein-based high-Ca diet actually became more obese and displayed higher WAT inflammation, compared to lower-Ca controls or mice fed high-Ca in a NFDM matrix. This differs from observations in high-Ca fed aP2-agouti transgenic mice fed an obesity-promoting diet where increased dietary Ca attenuated weight gain, adiposity, and WAT inflammation [5,20,21,34]. Experimental variables that may have contributed to these discrepancies include diet composition, energy consumption, genetic variation, and study duration.

In the present study, body weight gain was not significantly different between control mice and mice fed high-Ca when controlling for energy consumption as a covariate. Thus, increased energy consumption can explain differences in body weight gain between these diet treatment groups, whereas daily food intake was not different in aP2-agouti transgenic mice fed an obesigenic diet containing high Ca [5,20,21,34]. Similar to our results, 21 wk food consumption was increased in DIO male C57BL/6J mice fed high Ca in the context of a high fat diet (60% kcal as fat) containing whey, and a modest non-significant increase was observed in DIO mice fed a high Ca, casein-based diet [6]. Mice fed a 43% fat, NFDM-based high-Ca diet did not display a difference in energy intake relative to low Ca, casein controls [7]. Thus, results in polygenic DIO mouse models are consistent in that animals fed a high-Ca diet containing NFDM or whey protein display reduced body weight gain and adiposity despite increased or unchanged energy intakes. Protein source, and not Ca level, primarily contributed to body composition differences in DIO rats fed differing levels of Ca in the context of different dairy protein sources (NFDM, whey, or casein) [35]. These observations indicate that non-Ca components derived from dairy mainly drive adiposity reduction in polygenic DIO rodents.

Suppression of circulating calcitriol via increased Ca intake has been proposed as a primary mechanism for the anti-obesity and anti-inflammatory properties of high dietary Ca [22]. There is evidence that mice lacking the nuclear vitamin D receptor or enzyme required to generate calcitriol have reduced adiposity, lower serum leptin, and increased food intake [36]. Reduced adiposity in aP2-agouti transgenic mice fed obesigenic high-Ca diets was associated with reductions in blood calcitriol [20,21]. However, we observed that adiposity and body weight outcomes in DIO mice fed high-Ca in isolation and in high-Ca + NFDM showed no relation to differences in plasma calcitriol, indicating that changes in calcitriol are not sufficient to explain differences in body weight and adiposity in this model.

Mice that consumed high-Ca in a soy protein-based diet demonstrated increased feed efficiency, in contrast to a lower feed efficiency in mice fed high-Ca + NFDM. These results could emanate from diet-related differences in gut energy uptake and/or tissue thermogenesis. There was in fact increased net uptake of energy across the gut in mice fed the soy-based high-Ca diet. An observed increase in fecal energy loss in high-Ca + NFDM fed mice cannot explain their reduced feed efficiency, since increased energy intake in this group negated any difference in metabolizable energy compared to controls. With respect to thermogenesis, reduced feed efficiency in mice fed high-Ca + NFDM was not accompanied by increased BAT UCP1 protein expression. Since UCP1 expression is a surrogate for BAT thermogenic activation, one interpretation is that in the high-Ca + NFDM mice enhanced BAT thermogenesis did not contribute to the anti-obesity effects of this diet. Alternatively, BAT UCP1 expression was maintained at control levels despite lower body weight in the high-Ca + NFDM mice, so another interpretation is that this diet resulted in relative retention of BAT thermogenic potential. The increased BAT size and UCP1 protein expression in obese mice fed high-Ca was reminiscent of that seen in cafeteria fed rats [37], and this might be explained teleologically as a physiological adaptation to overnutrition. Additional studies of thermogenesis-related pathways in muscle and other tissues are warranted to clarify the mechanisms underpinning reduced feed efficiency in high-Ca + NFDM fed mice. The influence of calcitriol on WAT and muscle expression of IL-15 has been proposed to alter energy metabolism, with expression increased upon feeding high-Ca [20,26]. Increased IL-15 secretion has also been hypothesized to discourage lipid accumulation [20,38]. In our studies, WAT IL-15 gene expression was actually decreased in mice fed high-Ca compared to controls (a relationship held constant even after controlling body weight gain), and there were no significant differences in plasma IL-15 across diets. Thus, in polygenic DIO mice, differences in adiposity and feed efficiency were not associated with diet-related changes in WAT IL-15 expression or circulating IL-15 concentrations.

Non-Ca bioactive components of dairy thought to contribute to the anti-obesity effects of NFDM may be derived primarily from the whey protein fraction [22]. Supplementation with whey protein isolate reduced adiposity and increased energy expenditure in high fat fed mice [39]. Whey protein is a rich dietary source of branched chain amino acids (BCAA), which could in theory contribute to activation of thermogenesis. Leucine or whey protein isolate supplementation of drinking water increased energy expenditure and reduced diet induced obesity in mice without reducing energy intake [39,40]. Leucine when provided to muscle cell cultures increased energy consumption and indices of mitochondrial biogenesis (e.g., PGC-1 expression, mitochondrial mass), hypothesized to result from activation of mammalian target of rapamycin complex 1 (mTORC1) [41,42] or indirectly through anaplerosis [43]. Whether BCAA derived from the NFDM diet contributes to metabolic outcomes requires further experimental validation. Notably, milk proteins also possess bioactive peptides that have ACE-inhibitory properties [44], and the ACE product angiotensin II enhances adipocyte lipid storage, promotes inflammation via activation of the NFκB pathway, and stimulates ROS-mediated insulin resistance [45].

Chronic inflammation of WAT, hallmarked by macrophage accumulation, contributes to obesity-associated morbidities. Since calcitriol evokes a pro-inflammatory response in both adipocytes and macrophages, and enhances pro-inflammatory mediators in co-cultures of both cell types *in vitro *[19], high dietary Ca suppression of plasma calcitriol may have anti-inflammatory properties [20]. High dietary Ca both with and without dairy reduced WAT and systemic inflammation compared to low dietary Ca in aP2-agouti transgenic obese mice [21]. However, interpretations of these observations are confounded by reduced adiposity resulting from high dietary Ca intake in that model. We found that WAT expression of the pan-macrophage surface antigen CD68 correlated positively with body weight regardless of diet. WAT mRNA abundance for CD11d (an immune cell-specific cell adhesion molecule expressed on a subset of macrophages [46]) also correlated with body weight, but for any given weight was lower in NDFM-fed mice compared to controls or high-Ca fed mice. Diet-associated differences in most WAT inflammatory gene markers were lost when controlling for body weight. These results conclusively demonstrate that dietary Ca and/or NFDM effects on WAT inflammation in DIO mice are primarily explained by their impact on weight. In DIO mice fed different levels of fat (10%, 45%, and 60%), WAT macrophage marker expression correlated with body weight and adiposity in lean-to-moderately obese animals [24], similar to our current observations. These findings support the hypothesis that WAT macrophage infiltration (at least for some macrophage sub-types) is closely yoked to expansion of energy storage capacity during modest caloric overnutrition, possibly as a mechanism for macrophage-mediated WAT extracellular matrix and vascular remodeling to accommodate normal physiologic adipocyte hypertrophy. Significant correlations between WAT inflammatory markers and body weight was reported when looking across disparate mouse obesity models [15], and positive correlations were observed between %CD14+ cells in isolated stromal vascular cells from WAT and BMI in normal weight to obese humans [47,48]. Shaul et al. [49] have also considered a remodeling function based on dynamic M2-like macrophage phenotypes in rodents. The observations that WAT macrophage marker expression closely tracks body weight (at least in lean to moderate obese states) highlights a need to understand weight-specific immune signals and macrophage functions during normal, non-pathological WAT expansion and contraction.

## Conclusion

Mechanisms underlying the anti-obesity and/or anti-inflammatory properties of dairy components remain to be clarified. A primary role for increased dietary Ca is not supported in the current DIO mouse model since high Ca fed in a soy protein-based diet did not reduce, and in fact increased, obesity and associated adipose inflammation in stark contrast to animals fed high Ca in a NFDM-based matrix. Further deconvolution of the complex components of dairy foods is required in order to identify those specific factors that influence energy balance and inflammation.

## Abbreviations

ACE: angiotensin converting enzyme; BAT: brown adipose tissue; BCAA: branched chain amino acids; Ca: calcium; CGRP: calcitonin gene related peptide; DIO: diet-induced obese; FAS: fatty acid synthase; FIZZ: found in inflammatory zone; EPI: epididymal; HIF: hypoxia inducible factor; IL: interleukin; MCP: monocyte chemoattractant protein; NFDM: nonfat dry milk; ROS: reactive oxygen species; RP: retroperitoneal; SC: subcutaneous; TNF: tumor necrosis factor; UCP: uncoupling protein; WAT: white adipose tissue.

## Competing interests

The authors declare that they have no competing interests.

## Authors' contributions

All authors edited the manuscript, and read and approved the final version of the paper. APT and TND were jointly responsible for conducting the studies and sample assays. APT and SHA were primarily responsible for writing, JBD and PJO assisted with sample processing and data analysis.

## Supplementary Material

Additional file 1 **Quantitative PCR primer-probe information for metabolic and inflammatory gene targets**. Click here for file

Additional file 2 **Brown adipose tissue (BAT) protein content of uncoupling protein 1 (UCP1) in DIO mice fed 0.5% Ca (Control), 1.5% Ca (High-Ca), or 1.5% Ca + nonfat dry milk (High-Ca + NFDM)**. Values are means + SE, n = 8/treatment (arbitrary densitometry units). Treatments with different letters are significantly different (P < 0.05). UCP1 protein expression is normalized to glyceraldehyde 3-phosphate dehydrogenase (GAPDH) protein expression. Photo shows a representative Western blot result. Click here for file

Additional file 3 **Fecal weight (A), energy (B), and energy loss (C) in DIO mice fed 0.5% Ca (Control), 1.5% Ca (High-Ca), or 1.5% Ca + nonfat dry milk (High-Ca + NFDM)**. Values are means + SE, n = 10/treatment. Treatments with different letters are significantly different (P < 0.05). Click here for file
