# Supplementary material for: A high calcium diet containing nonfat dry milk reduces weight gain and associated adipose tissue inflammation in diet-induced obese mice when compared to high calcium alone
Source: Nutr Metab (Lond). 2012 Jan 23;9:3. doi: 10.1186/1743-7075-9-3 (PMC3284427; doi:10.1186/1743-7075-9-3)
Supplement: Additional file 1 — Quantitative PCR primer-probe information for metabolic and inflammatory gene targets. [file 1743-7075-9-3-S1.PDF]

Taqman Gene Expression Assays-on-Demand (Applied Biosystems)

Gene ID	Gene Name	Assay ID	Amplicon Size
Itgad	integrin alpha D (CD11d)	Mm01159115_m1	69
Il6	interleukin 6	Mm99999064_m1	75
Cd68	CD68 antigen	Mm03047339_m1	84
Ccl2	chemokine (C-C motif) ligand 2	Mm00441242_m1	74
Tnf	tumor necrosis factor (TNF-α)	Mm99999068_m1	63
Hif1a	hypoxia inducible factor 1, alpha subunit	Mm01283760_m1	71
Retnla	resistin like alpha (FIZZ-1)	Mm00445109_m1	86
Il10	interleukin 10	Mm00439614_m1	79
Il15	interleukin 15	Mm00434210_m1	73
Calca	calcitonin gene related polypeptide	Mm01274759_g1	164
Fas	fatty acid synthase	Mm00662319_m1	67
Ucp2	uncoupling protein 2	Mm00627597_m1	69
Hprt1	hypoxanthine guanine phosphoribosyl transferase 1	Mm00446968_m1	65
