# Supplementary figures and images for: A high calcium diet containing nonfat dry milk reduces weight gain and associated adipose tissue inflammation in diet-induced obese mice when compared to high calcium alone
Source: Nutr Metab (Lond). 2012 Jan 23;9:3. doi: 10.1186/1743-7075-9-3 (PMC3284427; doi:10.1186/1743-7075-9-3)

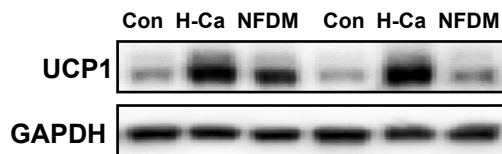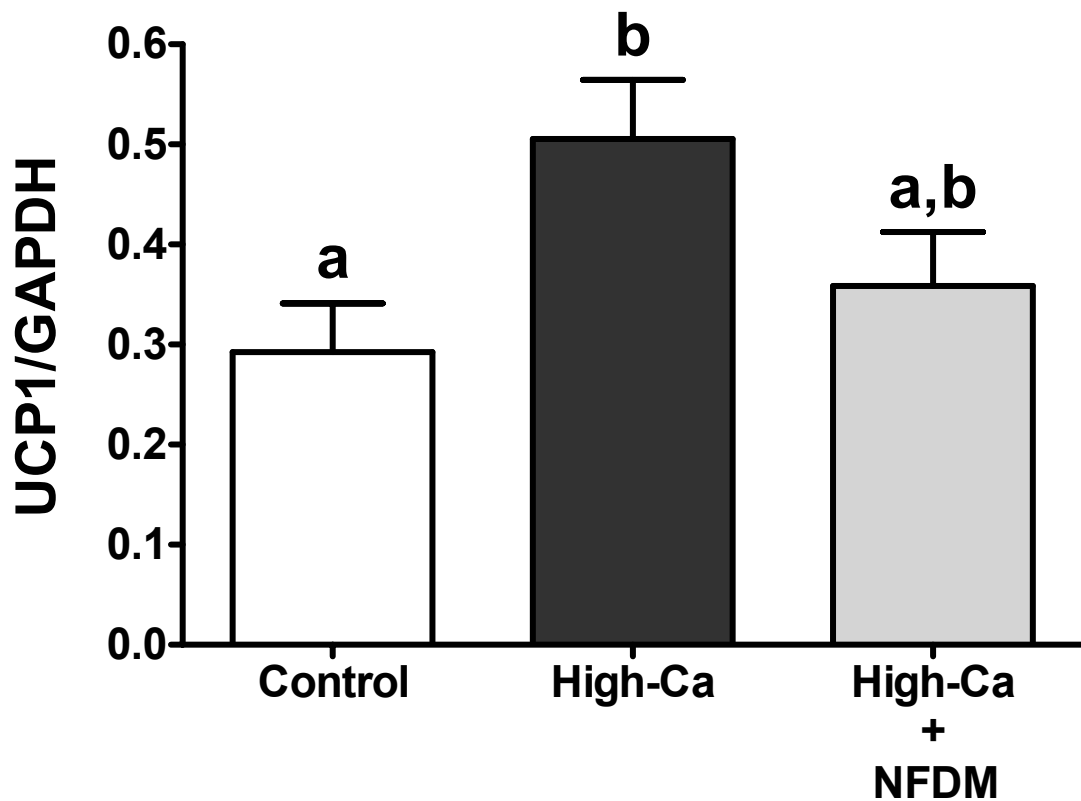

Supplement: Additional file 2 — Brown adipose tissue (BAT) protein content of uncoupling protein 1 (UCP1) in DIO mice fed 0.5% Ca (Control), 1.5% Ca (High-Ca), or 1.5% Ca + nonfat dry milk (High-Ca + NFDM). Values are means + SE, n = 8/treatment (arbitrary densitometry units). Treatments with different letters are significantly different (P < 0.05). UCP1 protein expression is normalized to glyceraldehyde 3-phosphate dehydrogenase (GAPDH) protein expression. Photo shows a representative Western blot result. [file 1743-7075-9-3-S2.PDF]

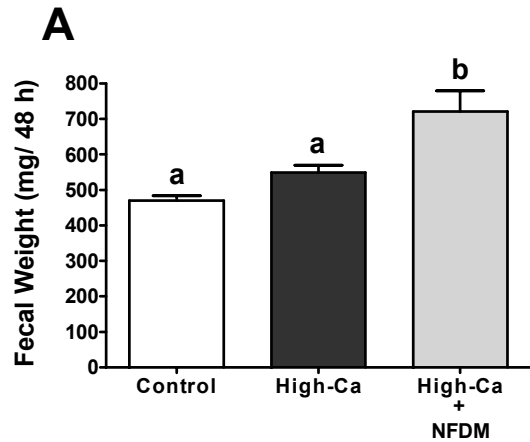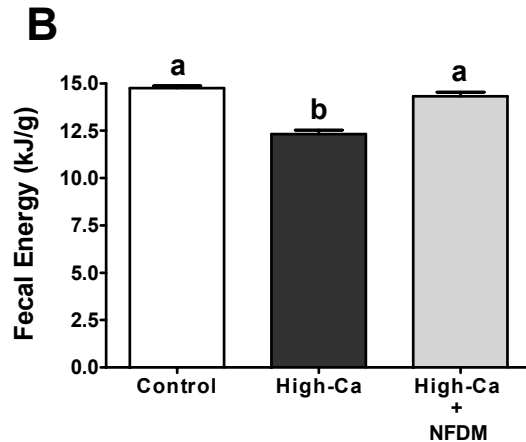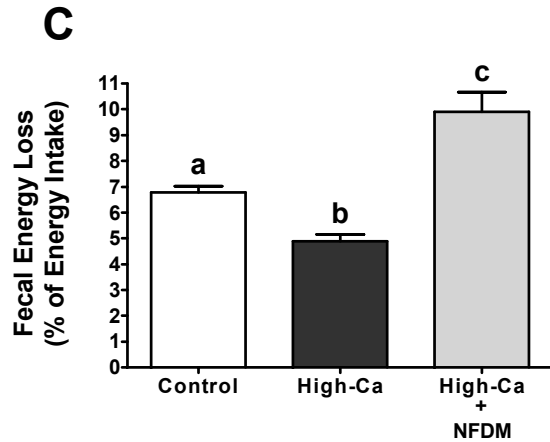

Supplement: Additional file 3 — Fecal weight (A), energy (B), and energy loss (C) in DIO mice fed 0.5% Ca (Control), 1.5% Ca (High-Ca), or 1.5% Ca + nonfat dry milk (High-Ca + NFDM). Values are means + SE, n = 10/treatment. Treatments with different letters are significantly different (P < 0.05). [file 1743-7075-9-3-S3.PDF]
